# Effect of simulated patient death on emergency worker’s anxiety: a cluster randomized trial

**DOI:** 10.1186/s13613-016-0163-3

**Published:** 2016-07-07

**Authors:** A. L. Philippon, J. Bokobza, B. Bloom, A. Hurbault, A. Duguet, B. Riou, Y. Freund

**Affiliations:** UPMC Univ-Paris 6, UMRS INSERM 1166, IHU ICAN, Paris Sorbonne Université, Paris, France; Emergency Department, Hôpital Pitié-Salpêtrière, Assistance Publique-Hôpitaux de Paris (APHP), Paris, France; Emergency Department, Barts Health NHS Trust, London, UK; Pneumology and Medical Intensive Care Unit, Hôpital Pitié-Salpêtrière, APHP, Paris, France

**Keywords:** Simulation, Simulated patient death, Anxiety, Emergency

## Abstract

**Objective:**

Simulation-based teaching offers promising and diverse teaching possibilities. We aim to assess whether the death of the manikin increased anxiety amongst learner compared to similar simulation-based course where the manikin stays alive.

**Methods:**

We conducted a cluster randomized study amongst multidisciplinary teams of emergency workers. Teams of physicians, nurses, and healthcare assistants were randomly assigned to participate in a simulation-based course where the simulated patient died (death group) or not (life group). We assessed anxiety at 1 month after the teaching using Spielberger STAI-state anxiety questionnaire. We compared reduction of anxiety when facing a life-threatening situation in both groups.

**Results:**

We included 25 teams for a total of 129 participants. We analysed 63 participants in the death group and 57 in the life group. Baseline characteristics were similar in both groups, including baseline anxiety (STAI-state score 39.6 (7.8) in the death group vs 38.6 (7.1) in the life group). We report a significant reduction in both groups 1 month after the training: 6.6 (7.8) vs 6 (8.0), mean difference 0.5 (−2.4; 3.4). At 3 months, we report a significant greater reduction of anxiety in the death group (mean difference 4 [0.1; 7.9]).

**Conclusion:**

We observed in our sample that unexpected simulated patient death did not increase anxiety amongst multidisciplinary emergency workers.

## Background

Simulation-based training is an effective tool for the training of healthcare professionals and students. It is effective in enhancing knowledge, skills, and behaviour in life-threatening situations (LTSs) [1–7]. One of the key applications of simulation is in practising the management of patients with high clinical acuity, such as LTS, when prognosis is highly dependent on immediate actions and decisions. During such sessions, participants are trained in an environment in which they can make mistakes which have no effect on live patients. However, the situation may be no less stressful than one with a live patient [8–10].

The death of a simulated patient remains controversial because it may be perceived as a consequence of medical error and as such may endanger the safe learning environment created for trainees [11]. However, during professional life, healthcare providers often encounter LTS (such as cardiac arrests), and the subsequent patient death. The reluctance of the simulation trainer to allow the simulated patient to die during simulation-based training is not supported by scientific data, but rather by a general consensus and expert opinion [11]. The death of a simulated patient as an educational episode offers valuable teaching opportunities by reproducing a highly stressful situation that doctors are likely to experience in their usual practice. This may allow learners to develop their practice surrounding situations involving patient death, for example enhancement of communications skills with colleagues, patients, and their families [12–14]. However, the risk and benefits of unexpected death of the manikin are unclear and debated. One argument is that simulation-based learning should not be a punitive experience, nor have negative emotional effects on learners [11, 15]. In contrast, it is suggested that learners may benefit from learning from “errors”; from accepting that some patients’ deaths are inevitable regardless of any medical management; and from being exposed to higher emotional stress levels during their simulated training [16]. Only one randomized study reported immediate post-training negative emotions amongst those facing manikin death [17], but to our knowledge, there are no data on learners’ anxiety and the long-term emotional effect of unexpected death is unknown. Furthermore, although cardiac arrests are usually managed by several healthcare professionals in the hospital (physicians, nurses, and healthcare assistants), no previous studies reported the effect on multiprofessional teams of unexpected manikin death in a simulation-based training.

We tested the hypothesis that the death of a simulated patient does not increase anxiety within a healthcare team. We aimed to assess the impact of simulation-based training with unexpected simulated patient death on the learners’ anxiety when facing life-threatening situation.

## Methods

### Study design

We conducted a prospective cluster randomized trial on multidisciplinary teams that work in an emergency department (ED) in Paris, France. Each team was randomized before the simulation session into a life group or in a death group. Randomization was performed using the “RANDOM” function of MS Excel (Microsoft, Redmond, WA, USA). Participants were blinded about study objectives and were advised that they were participating in a study designed to assess emotions whilst managing life-threatening situations. We followed CONSORT guidelines for the reporting of cluster randomized trial.

### Objective and endpoints

Our primary objective was to study the effect of a simulated patient’s unexpected death on the change in learners’ anxiety caused by a life-threatening situation. We also studied participants’ feeling and appreciation of the training. We anticipated that the simulation-based courses will decrease anxiety amongst participants when facing a LTS regardless of group allocation. Our primary endpoint was the change in anxiety when facing a LTS between baseline and 1 month after the training. Secondary endpoints include satisfaction regarding the training and willingness to undertake further similar simulation-based courses. We also studied 3-month anxiety reduction.

We evaluated anxiety through the validated French version of the Spielberger state part of the State-Trait Anxiety Inventory (STAI-S) [18]. The state elements of the score measures the transitional emotional status evoked by a stressful situation (namely caring for a patient with a LTS), using 20 items each rated by a 4-point Likert scale (possible score range 20–80). Higher scores are positively correlated with higher levels of anxiety. A score >37 for men and >42 for women reflects high anxiety, and a score >48 for men and >55 for women corresponds to anxiety liable to interfere with performance [19]. The level of anxiety was evaluated in all subjects before the training and then assessed at 1 and 3 months using the state elements of the STAI. We also assessed baseline anxiety of subjects using the trait elements of the STAI. The state element of STAI assess the anxiety resulting from a situation or confrontation, whereas the trait element may be seen as an assessment of “baseline anxiety,” the anxiety caused by normal situation in day-to-day life [20]. These measures have been reported to have excellent interrater agreement for their assessment and good correlation with previously used methods to assess anxiety [19–24].

Electronic questionnaires were submitted to all participants to record anonymously their feeling and feedback of the training on a numeric scale that ranges from 0 (lower) to 10 (higher).

### Selection of participants

Participants were physicians, medical students, nurses, and healthcare assistants employed in EDs in four academic hospitals in Paris, France. Physicians and medical students were enrolled from all four study sites. Nurses and healthcare assistants were enrolled from Pitie-Salpetriere hospital, where every nurse and healthcare assistant had a mandatory course of multiprofessional simulation-based training. We enrolled participants in this context, with informed consent. Participants that refused to participate and those with no follow-up at 1 month were excluded. We included participants during a 4-month period, from March to June of 2015. Each simulation team included a core consisting of one resident, two nurses and one healthcare assistant. To include all volunteers and keep the core composition, some teams included a fifth participant (healthcare assistant or medical student). Our institutional review board (Comite de protection des personnes—Paris Ile de France 6, Paris, France) authorized the study as it involved no patients. The Ethics Committee of the French society of intensive care (SRLF, Paris, France) approved the study. All subjects were followed up with a face-to-face interview and anonymous electronic questionnaire at 1 and 3 months.

### Description of the training

All subjects participated in a simulation-based training (SBT) that comprised pre-briefing, scenario, and debriefing. During the pre-briefing, subjects were told that they were participating in a study investigating emotional responses in the settings of life-threatening situations. The scenario included a 35-year-old man in the ED with ventricular fibrillation subsequent to a Brugada syndrome. We randomly assigned each team in two groups: after three electrical shocks, the patient returns in spontaneous cardiac activity (life group), or the patient ends in asystole (death group). In the death group, an instructor had the role of the intensivist that admitted the patient to ICU for ongoing advanced life support. Participants were told immediately after the 10- to 15-min session that the patient underwent 45 min of unsuccessful advanced life support and was declared dead. During the debriefing, participants were encouraged to talk about the patient’s death. The pre-specified educational objectives of our scenarios were: to identify cardiac arrest, to initiate and provide basic life support then advanced life support (including calling the intensivist). Our non-technical skills educational objectives were mainly teamwork communication. We use a Laerdal low-fidelity manikin to practise simulation, with audio–video recording and debriefing (Laerdal, Stavanger, Norway, and Sim Station, Wien, Austria).

### Statistical analysis

Continuous data are expressed as mean (standard deviation—SD) when normally distributed and as median [25–75 interquartile range—IQR] if not. Categorical variables are expressed as number (percentage). Normality was tested using the Kolmogorov–Smirnov test. Comparison of data was made using Student’s t test and presented as mean differences and their 95 % exact confidence interval (CI) that included the analysis of the primary endpoint of change in STAI score between life group and death group. The reduction in STAI (score at day 0 minus score at 1 or 3 months) was compared using the paired Student’s t test for comparison of paired variable. Categorical variables were compared using Chi-square test or Fisher exact test when appropriate.

With no pre-existing literature or pilot study on this subject, we evaluated the first 35 included participants and found a mean reduction of five points for the primary endpoint [25]. With a non-inferiority a priori hypothesis, we estimated that we needed to report a lower bound of the 95 % CI of the primary endpoint in the death group that should not be less than 3 points below the mean reduction in the life group. With a hypothesis of a mean reduction in anxiety of 5 in the life group, the lower bound of the 95 % CI of the mean reduction in anxiety in the death group should be higher than 2. With a mean cluster size of 4, an alpha of 2.5 %, a power of 90 % and accounting for an intraclass correlation of 0.01, we calculated that we needed 60 subjects in each group (PASS 14, Statistical Solution Ltd, Cork, Ireland).

## Results

### Description of the population

Twenty-five teams of learners accounting for 129 subjects participated in our simulation-based training, of which we included 120 in our study (three refused to participate, and data were incomplete for six of them). There were 30 healthcare assistants, 52 nurses, 14 medical students, and 24 residents. We randomly assigned 13 teams (63 participants) in the death group and 12 (57 participants) in the life group. The mean age of participants was 30 years (SD 7) and 34 (28 %) were men. The two groups had similar characteristics (Table 1). Baseline anxiety levels were similar between the two groups, with a mean STAI-trait score of 39.6 (7.8) in the death group vs 38.6 (7.1) in the life group (mean difference of 2.5, 95 % CI [−1.2; 6.1]). We also report a similar STAI-state score in both groups for the anxiety caused by a LTS before the course: 46.8 (11.2) in the death group vs 44.3 (9.7) in the life group (mean difference 0.99 [−1.7; 3.7]).

**Table 1:** Baseline characteristics of participants

Characteristic	All participants	Death group	Life group
N	120	63	57
Age, mean (SD)	30 (7)	30 (6)	29 (8)
Sex male	34 (28 %)	20 (32 %)	14 (25 %)
Occupation			
Healthcare assistant	30 (25 %)	18 (29 %)	12 (21 %)
Nurse	52 (43 %)	26 (41 %)	26 (44 %)
Medical student	14 (12 %)	6 (10 %)	8 (14 %)
Resident	24 (20 %)	13 (21 %)	11 (20 %)
Have previously participated in			
Simulation course	96 (80 %)	47 (75 %)	49 (86 %)
Cardiac arrest situation	82 (69 %)	47 (75 %)	35 (63 %)
STAI			
State part, mean (SD)	45.6 (10.1)	46.8 (11.2)	44.3 (9.7)
Trait part, mean (SD)	39.1 (7.0)	39.6 (7.8)	38.6 (7.1)

*SD* standard deviation, *STAI* Spielberger Trait Anxiety Inventory

### Endpoints analysis

**Fig. 1:**
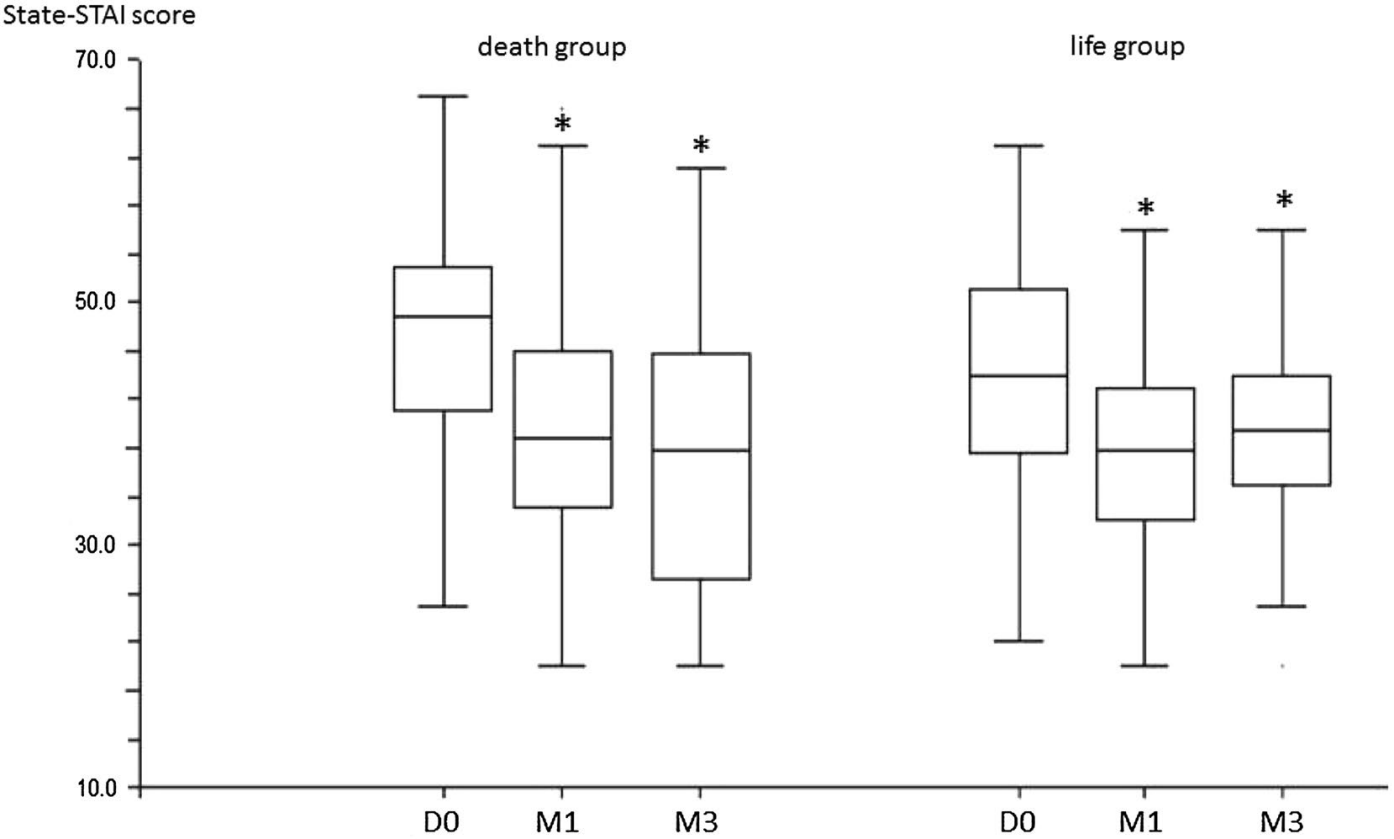
Comparison of STAI-state score between death group and life group. Data are expressed as *box plot* (median, interquartile range, and outliers) *p < 0.05 compared to day 0. *M1* after 1 month; *M3* after 3 months

Figure 1 shows the reduction in anxiety in both groups. There was no significant difference in the primary endpoints between the two groups: the mean difference between the change in STAI-state anxiety score from baseline to one month was 6.6 (7.8) in the death group vs 6.0 (8.0) in the life group (mean difference between groups 0.5 [−2.4; 3.4]) (Table 2).

**Table 2:** Comparisons of anxiety levels between groups

	All patients	Death group	Life group	Mean difference (death group–life group)	95 % CI
Baseline STAI-trait	39.1 (7.0)	39.6 (7.8)	38.6 (7.1)	0.1	[−1.7; 3.7]
Baseline STAI state	45.6 (10.0)	46.8 (11.2)	44.3 (9.7)	2.5	[−1.2; 6.1]
STAI state at M1	39.3 (9.2)	40.2 (10.1)	38.3 (8.0)	2	[−1.3; 5.3]
STAI state at M3	38.1 (10.1)	37.1 (11.1)	39.1 (8.3)	−1.8	[−5.7; 2.1]
Reduction in STAI state					
Between baseline and M1	6.3 (7.9)	6.6 (7.8)	6 (8.0)	0.5	[−2.4; 3.4]
Between baseline and M3	7.7 (10.3)	9.5 (10.3)	5.6 (9.9)	4	[0.1; 7.9]

*STAI* State-Trait Anxiety Inventory, *CI* confidence interval, *M1* 1 month, *M3* 3 months

Global satisfaction and willingness to undertake another simulation-based course was very high and similar in the two groups [respectively ranked at 10 (8; 10) and 8 (7; 9) in both groups on a 1–10 scale]. Of note, the simulation-based session lasted slightly longer in the death group (880 vs 853 s, mean difference 29 [15; 42]) and debriefing duration was shorter (31 vs 34 min, mean difference −3 [−5; −1]). At 3 months, we show a sustained reduction in the anxiety caused by a LTS with a mean reduction of 7.7 (10.3). Amongst the 107 subjects analysed at 3 months (seven were lost to follow-up), we report a significant greater reduction in the death group: 9.5 (10.3) vs 5.6 (9.9), mean difference 4.0 (0.1; 7.9).

## Discussion

In this cluster randomized trial, we randomly assigned teams of ED staff to treat a simulated patient that either dies or does not die during simulation training sessions. We hypothesized that unexpected simulated patient death was not associated with increased anxiety. We observed that training on simulated patients with LTSs substantially decreased anxiety levels of ED workers (whether trainees, nurses, or healthcare assistants). Under the hypothesis of a mean reduction of 5 in the control group and a delta of 3, our results confirm the non-inferiority hypothesis. This trial is the second that prospectively randomizes learners to see their manikin die or not.

Controversy remains on whether the unexpected death of a simulated patient is an option in simulation-based medical education. Rodgers et al. [26] advise that professionals have to be prepared to face patients’ death. Training on manikin’s death could help develop specific skills, including improved communication with families, and could help healthcare staff to anticipate their own feelings in a real-life clinical context [12]. This could also be valuable for students who’s curricula in France lack this experiential learning [27], and who may face a lack of support when treating patients with LTS during their training in hospital [28]. Two main issues may contribute to a reluctance to design simulation scenarios in which the patient dies. Firstly, it is suggested that learners should evolve in a safe learning environment and that a setting in which a simulated patient can die is not educationally safe. The death of the simulated patient could be perceived as potentially traumatizing for the learners. The second issue is that the death of a simulated patient may pass a threshold of cognitive load, after which learning outcomes are more poorly achieved [17].

We believe that learners should be trained to manage the death of their patients, as this will inevitably occur during their future occupation. As highlighted by Bruppacher et al. [11], this training must be performed in a non-punitive culture, with interprofessional collaboration and sophisticated debriefing. Our trial was designed to specifically address this matter and evaluate whether unexpected death during simulation-based training could actually help learners to deal with life-threatening situation. Our results show that these sessions helped in reducing ED workers anxiety, whether the manikin died or not. We do not report any significant difference in effect in the death group. Furthermore, at 3 months, participants in the death group had a greater reduction in anxiety compared to the control group. This suggests that training on a simulated patient that eventually dies helps prepare for this.

To our knowledge, this is the first study that evaluates the anxiety caused by the death of a simulated patient. The strength of this study lies in its design to address this specific question, and our results suggest a greater anxiety reduction even after 3 months. However, we did not address any cognitive effect or improvement as did Fraser et al. [17]. We believe that our results are very important for future literature in this field as we used a validated scale, which was absent in Fraser et al. study. Future research should identify whether death of a simulated patient may assist in achieving learning outcomes. We believe that some specific skills (as communication with the family, dealing with death with colleagues, etc.) can only be taught utilizing this modality and that since anxiety does not seem to be increased with this, simulated patient death can be considered.

## Limits

Our study presents some limitations. Firstly, this study was designed to address the anxiety caused by a LTS amongst healthcare workers at 1 and 3 months after the simulation. We did not evaluate immediate and short-term effect of simulated patient death. Moreover, we did not evaluate the cognitive impact and educational added value of the simulated patient death. Secondly, we did not assess the impact of real-life situations that happened between baseline and 1 month. It is possible that some participants had experienced real LTS in this time frame and that could have influenced the primary endpoint. Lastly, we did not study the effect of this intervention depending on the occupation of the participant, nor on their baseline STAI. We can imagine that the simulated patient death may have a different effect on participant with pre-existing high level of anxiety than on others, as it is possible that healthcare assistant, nurse, and physician may not have same impact of this simulation course. We found no significant difference in various sensitivity analyses; however, the small number of participants translated in a weak power to detect significant difference amongst subgroups.

## Conclusion

We confirmed that simulation-based course on LTS helped to reduce anxiety amongst multidisciplinary teams of ED workers. In our cluster randomized trial, we found that the death of the simulated patient resulted in similar anxiety reduction for the management of LTS in the emergency department.

## References

[1] Cook DA, Hatala R, Brydges R (2011). Technology-enhanced simulation for health professions education: a systematic review and meta-analysis. JAMA.

[2] Lorello GR, Cook DA, Johnson RL, Brydges R (2014). Simulation-based training in anaesthesiology: a systematic review and meta-analysis. Br J Anaesth.

[3] Issenberg SB, McGaghie WC, Petrusa ER, Lee Gordon D, Scalese RJ (2005). Features and uses of high-fidelity medical simulations that lead to effective learning: a BEME systematic review. Med Teach.

[4] Johnson RL, Cannon EK, Mantilla CB, Cook DA (2013). Cricoid pressure training using simulation: a systematic review and meta-analysis. Br J Anaesth.

[5] Kuduvalli PM, Jervis A, Tighe SQM, Robin NM (2008). Unanticipated difficult airway management in anaesthetised patients: a prospective study of the effect of mannequin training on management strategies and skill retention. Anaesthesia.

[6] Bruppacher HR, Alam SK, LeBlanc VR (2010). Simulation-based training improves physicians’ performance in patient care in high-stakes clinical setting of cardiac surgery. Anesthesiology.

[7] Scavone BM, Toledo P, Higgins N, Wojciechowski K, McCarthy RJ (2010). A randomized controlled trial of the impact of simulation-based training on resident performance during a simulated obstetric anesthesia emergency. Simul Healthc J Soc Simul Healthc.

[8] Hunziker S, Semmer NK, Tschan F, Schuetz P, Mueller B, Marsch S (2012). Dynamics and association of different acute stress markers with performance during a simulated resuscitation. Resuscitation.

[9] Bong CL, Lightdale JR, Fredette ME, Weinstock P (2010). Effects of simulation versus traditional tutorial-based training on physiologic stress levels among clinicians: a pilot study. Simul Healthc J Soc Simul Healthc..

[10] Andreatta PB, Hillard M, Krain LP (2010). The impact of stress factors in simulation-based laparoscopic training. Surgery.

[11] Bruppacher HR, Chen RP, Lachapelle K (2011). First, do no harm: using simulated patient death to enhance learning?. Med Educ.

[12] Yardley S (2011). Death is not the only harm: psychological fidelity in simulation. Med Educ.

[13] Truog RD, Meyer EC (2013). Deception and death in medical simulation. Simul Healthc J Soc Simul Healthc..

[14] Tan A, Ross SP, Duerksen K (2013). Death is not always a failure: outcomes from implementing an online virtual patient clinical case in palliative care for family medicine clerkship. Med Educ Online.

[15] Gaba DM (2013). Simulations that are challenging to the psyche of participants: how much should we worry and about what?. Simul Healthc J Soc Simul Healthc.

[16] Demaria S, Bryson EO, Mooney TJ (2010). Adding emotional stressors to training in simulated cardiopulmonary arrest enhances participant performance. Med Educ.

[17] Fraser K, Huffman J, Ma I (2014). The emotional and cognitive impact of unexpected simulated patient death: a randomized controlled trial. Chest.

[18] Spielberger CD, Gorsuch RL, Lushene R, Vagg PR, Jacobs GA. Manuel de L’inventaire D’anxiété État-Trait Forme Y (STAI-Y) (Bruchon-Schweitzer M, Paulhan I, Adapt. Française). Editions du Centre de Psychologie Appliquée. Paris; 1993.

[19] Spielberger CD (1983). Manual for the State-Trait Anxiety Inventory.

[20] Spielberger CD (1989). State-Trait Anxiety Inventory: a comprehensive bibliography.

[21] Stanley MA, Beck JG, Zebb BJ (1996). Psychometric properties of four anxiety measures in older adults. Behav Res Ther.

[22] Schmidt M, Freund Y, Alves M (2014). Video-based feedback of oral clinical presentations reduces the anxiety of ICU medical students: a multicentre, prospective, randomized study. BMC Med Educ.

[23] Marteau TM, Bekker H (1992). The development of a six-item short-form of the state scale of the Spielberger State-Trait Anxiety Inventory (STAI). Br J Clin Psychol.

[24] Wrenn KC, Mostofsky E, Tofler GH, Muller JE, Mittleman MA (2013). Anxiety, anger, and mortality risk among survivors of myocardial infarction. Am J Med.

[25] Philippon A-L, Bokobza J, Hurbault A (2015). 383 the death in simulation randomized trial: effect of simulated patient death on emergency worker’s anxiety. Ann Emerg Med.

[26] Rogers G, de Rooy NJ, Bowe P (2011). Simulated death can be an appropriate training tool for medical students. Med Educ.

[27] McIlwaine L, Scarlett V, Venters A, Ker JS (2007). The different levels of learning about dying and death: an evaluation of a personal, professional and interprofessional learning journey. Med Teach.

[28] Rhodes-Kropf J, Carmody SS, Seltzer D (2005). “This is just too awful; I just can’t believe I experienced that [horizontal ellipsis]”: medical students’ reactions to their “most memorable” patient death. Acad Med Featur Top End-Life Care.

